# Getting ready for host invasion: elevated expression and action of xyloglucan endotransglucosylases/hydrolases in developing haustoria of the holoparasitic angiosperm *Cuscuta*

**DOI:** 10.1093/jxb/erv482

**Published:** 2015-11-11

**Authors:** Stian Olsen, Bernd Striberny, Julien Hollmann, Rainer Schwacke, Zoë Popper, Kirsten Krause

**Affiliations:** ^1^Department of Arctic and Marine Biology, Faculty of Biosciences, Fisheries and Economics, UiT The Arctic University of Norway, Dramsveien 201, 9037 Tromsø, Norway; ^2^Institute of Botany, Christian-Albrechts-University of Kiel, Olshausenstrasse 40, D-24098 Kiel,Germany; ^3^Botany and Plant Science and Ryan Institute for Environmental, Marine and Energy Research, National University of Ireland Galway, Galway, Ireland; * Present address: ArcticZymes AS, Sykehusveien 23, 9019 Tromsø, Norway.; ^†^ Present address: Institute of Bio- and Geosciences (IBG-2: Plant Sciences), Forschungszentrum Jülich, Wilhelm-Johnen-Straße, D-52428 Jülich, Germany.

**Keywords:** Cell wall, *Cuscuta*, haustorial gene expression, haustorium development, parasitic plant, xyloglucan endotransglucosylase/hydrolase (XTH).

## Abstract

Changes in cell walls have been previously observed in the mature infection organ, or haustorium, of the parasitic angiosperm *Cuscuta*, but are not equally well charted in young haustoria. In this study, we focused on the molecular processes in the early stages of developing haustoria; that is, before the parasite engages in a physiological contact with its host. We describe first the identification of differentially expressed genes in young haustoria whose development was induced by far-red light and tactile stimuli in the absence of a host plant by suppression subtractive hybridization. To improve sequence information and to aid in the identification of the obtained candidates, reference transcriptomes derived from two species of *Cuscuta*, *C. gronovii* and *C. reflexa*, were generated. Subsequent quantitative gene expression analysis with different tissues of *C. reflexa* revealed that among the genes that were up-regulated in young haustoria, two xyloglucan endotransglucosylase/hydrolase (XTH) genes were highly expressed almost exclusively at the onset of haustorium development. The same expression pattern was also found for the closest XTH homologues from *C. gronovii*. *In situ* assays for XTH-specific action suggested that xyloglucan endotransglucosylation was most pronounced in the cell walls of the swelling area of the haustorium facing the host plant, but was also detectable in later stages of haustoriogenesis. We propose that xyloglucan remodelling by *Cuscuta* XTHs prepares the parasite for host infection and possibly aids the invasive growth of the haustorium.

## Highlight

Expression of cell wall-related genes marks the onset of haustorium development in the parasitic plant *Cuscuta*. Action assays suggest a central role for xyloglucan endotransglucosylases/hydrolases in host plant infection.

## Introduction

**Fig. 1.**
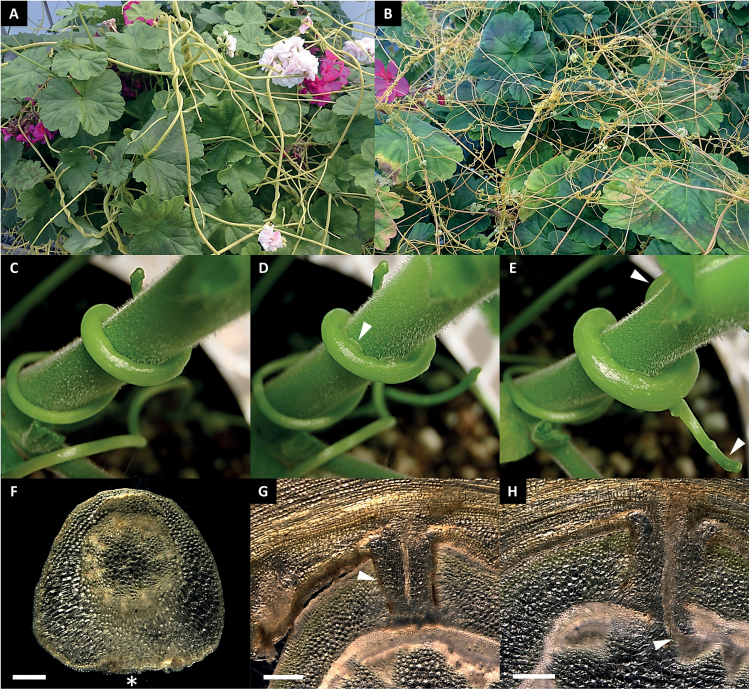
*Cuscuta* infecting the compatible host plant *P. zonale*. (A) *C. reflexa* and (B) *C. gronovii* parasitizing *P. zonale*. The infection of *P. zonale* by *C. reflexa* can be divided into three stages. (C and F) In the first swelling stage the parasite stem grows in width at the side facing the host (asterisk). (D and G) After attachment to the host surface (arrowhead in D), the haustorium (arrowhead in G) invades the host tissue (penetrating stage). (E and H) The mature stage is defined by established connections between the vascular systems of the two plants (arrowhead in H) and is recognized by apical shoot growth and the formation of additional side shoots (arrowheads in E). Cross-sections were made with respect to orientation of the parasite shoot axis in (F), and with respect to the host shoot axis in (G) and (H). Scale bars are 500 µm.

The genus *Cuscuta* of the Convolvulaceae family (Solanales) includes ~200 species of thread-like parasitic plants with worldwide distribution (Dawson *et al.*, 1994; Garcia *et al.*, 2014). Being devoid of proper leaves and roots, and exhibiting very little to no photosynthetic activity, *Cuscuta* spp. are dependent on parasitizing a host plant to survive and reproduce. Host attachment and intrusion of stem and leaf tissue are mediated by specialized infection organs called haustoria that develop close to the apical stem tip. The initiation of haustorium differentiation is marked by site-specific cell elongation in areas where the parasite has contact with a host plant, and which is visible as a unilateral swelling of the parasite’s stem (Fig. 1). Subsequently, adhesive substances are secreted by the epidermis around the protruding haustorium (Vaughn, 2002), anchoring the parasite to the host and allowing the infection organ to grow into the host tissue using a combination of mechanical pressure and enzymatic digestion (Nagar *et al.*, 1984; Johnsen *et al.*, 2015; Kaiser *et al.*, 2015) (Fig. 1). Upon reaching host xylem or phloem elements, searching hyphae emerge from the body of the haustorium and differentiate into these respective cell types, facilitating the transport of water and sugars from host to parasite (Vaughn, 2006). The successful connection to the host’s nutritional resources is visibly indicated by further swelling of the attached region, side shoot protrusion from the infection site, and restoration of apical tip growth, which typically ceases during the two previous stages (Fig. 1). Through the haustorium, a large variety of compounds are taken up by *Cuscuta* including small inorganic and organic molecules such as sugars, hormones, and amino acids, and macromolecules such as proteins and RNAs. These appear to serve both nutritional and regulatory purposes in this mutual interaction (Aly, 2013; Kim and Westwood, 2015). Having to share so many resources exposes the parasitized plants to a considerable amount of stress, and can result in reduced biomass or even host death in cases of severe attacks. In agricultural areas, the control of parasitic plants is difficult due to the physiological similarity and intimate connections of host and parasite. Thus, increased knowledge of the molecular mechanisms underlying haustorium development and host infection is required to develop effective strategies for combating the negative impacts of parasitic weeds (Aly, 2007; Alakonya *et al.*, 2012; Jiang *et al.*, 2013; Ichihashi *et al.*, 2015). This is even more adamant when considering that many species of *Cuscuta*, in contrast to most other parasitic lineages, are generalists, being able to parasitize a large number of dicotyledonous plant species from different phylogenetic taxa. This variation in hosts can entail some molecular tuning of the infection process, for example by adapting the type of nutrient transfer cells in the mature haustoria to the type of host (Christensen *et al.*, 2003). The onset of haustorium differentiation, however, does not seem to be influenced by different hosts, but rather depends on the presence of general signals initiating the infection process. The fact that the formation of *Cuscuta* haustoria can be stimulated in the absence of a host plant by applying a combination of far-red (FR) light and tactile stimuli corroborates this hypothesis (Tada *et al.*, 1996). Haustoria induced in this way share morphological traits with haustoria developing in contact with a host plant, but have the advantage that the early stages of their development are more uniform and predictable than when induced by host attachment. In addition, in molecular studies, the consideration of RNA molecules transferred from the host can be neglected.

Cell wall polymer profiling in infecting and non-infecting tissues of *Cuscuta* recently revealed such substantial differences in cell wall composition (Johnsen *et al.*, 2015) that the initiation of changes to the cell wall must be assumed to occur early in haustoriogenesis. In the present study, gene expression in haustoria and stems of two *Cuscuta* species, *C. reflexa* and *C. gronovii*, was therefore analysed with the goal of identifying cell wall-related genes that mark the onset and progress of haustorium development. *Cuscuta reflexa*, a member of the subgenus *Monogynella*, possesses thick and yellow to green stems, while *C. gronovii* has thinner and more delicate stems of yellow to orange colour (Fig. 1A, B) and belongs to the subgenus *Grammica* (Costea *et al.*, 2015). The distinct expression of xyloglucan endotransglucosylases/hydrolases (XTHs) in young haustorial tissues of both *C. reflexa* and *C. gronovii* was further substantiated by protein immunolocalization and xyloglucan endotransglucosylation (XET) assays. The potential function of XTHs in *Cuscuta* haustorium development and host plant infection will be discussed.

## Materials and methods

### Plant material

*Cuscuta reflexa* and *C. gronovii* were propagated on the compatible host *Pelargonium zonale* (Fig. 1) in a greenhouse at the Phytotron of the University of Tromsø, Norway, in 24h of light at 21 °C. For RNA sequencing and for the induction of haustorium development with the FR light system, filaments of *Cuscuta* that were distal to the host infection sites were used. For the analysis of host-induced haustorial stages, infection sites on *P. zonale* were visually inspected for the characteristics of the different stages: the swelling stage before penetration of the host that was defined as the starting point of haustoriogenesis (Fig. 1C, 1F); the penetrating stage after initial intrusion into the host tissue but before the parasite is able to feed (Fig. 1D, 1G); and the mature feeding stage that is recognized by further swelling of the attached region, protruding side shoots from the infection site, and restoration of apical tip growth (Fig. 1E, 1H). Other samples encompassed stems at least 5cm distal to infection sites and to shoot tips, elongating stem regions (an area of 3–5cm below the apical tip), and shoot tips (first apical centimetre of shoot).

### FR light induction of haustoriogenesis

Host-free induction of haustorium development was carried out as described by Tada *et al.* (1996) with minor modifications. To facilitate tactile stimuli, apical shoot tips of ~10cm (harvested from the propagating culture) were placed between two plastic Petri dishes (Ø=13.5cm) that were lightly pressed and held together using adhesive tape. The pressed shoot tips were then placed in an upright position and irradiated with FR light (740nm) for 1h. After light treatment, the shoots were kept in darkness before further analysis. Tissue for RNA isolation was harvested from stem segments with and without haustoria, 3 d or 6 d after FR light induction, as indicated.

### RNA isolation

All plant material used for RNA isolation was snap-frozen in liquid nitrogen and homogenized using a TissueLyser (Qiagen, Hilden, Germany). Total RNA was isolated using a combination of the hot borate method (Wan and Wilkins, 1994), and phenol–chloroform extraction in which pre-warmed (65 °C) borate buffer (200mM Borax, 30mM EDTA, 1% (w/v) SDS) and phenol were added to the frozen plant material to make up the first liquid–liquid extraction. Subsequently, one extraction with phenol:chloroform:isoamylalco hol (25:24:1) and two with chloroform:isoamylalcohol (24:1) were executed before total RNA was precipitated in 2M LiCl at 4 °C overnight. In order to remove residual DNA, the RNA samples were treated with DNase using the DNA-*free* kit (Ambion Inc., Austin, TX, USA). Removal of DNA and integrity of RNA were checked by agarose gel electrophoresis.

### Construction of suppression subtractive hybridization (SSH) libraries and sequencing of subtracted cDNA clones

The two cDNA libraries used for generating differentially expressed SSH libraries were synthesized from 250ng of DNase-treated total RNA isolated from FR light-induced haustoria or stems 3 d after light treatment (for each RNA isolation, material was pooled from six induced shoots) using the SMARTer Pico PCR cDNA Synthesis kit (Clontech, Mountain View, CA, USA). SSH was carried out with the PCR-Select cDNA Subtraction Kit (Clontech). The Advantage 2 PCR Kit (Clontech) was used for all PCR amplifications. After amplification, the differentially expressed cDNAs were cloned into the pGEM-T Easy vector system (Promega, Madison, WI, USA). One Shot TOP10 Chemically Competent *Escherichia coli* cells (Invitrogen, Carlsbad, CA, USA) were transformed with the SSH libraries and incubated on LB/Carbenicillin/X-Gal/IPTG plates at 37 °C for blue/white screening. All procedures were carried out according to the manufacturers’ instructions. Plasmid DNA was isolated from white colonies by alkaline lysis (Birnboim and Doly, 1979) and sequenced with M13F primer (5′ GTAAAACGACGGCCAGT 3′) by Sanger sequencing (Macrogen Korea, Seoul, Korea).

### De novo *transcriptome sequencing and assembly*

Poly(A)^+^ RNA purification, reverse transcription, size fractionation, titration, and sequencing were performed at the Norwegian High Throughput Sequencing Centre (NSC; Oslo, Norway) on a Roche GS FLX (454) sequencer. One-eighth of a run for both libraries was done as a test run, followed by re-titration and a full run with the same libraries. Together, both runs yielded 945 454 raw reads for *C. reflexa* and 914 135 raw reads for *C. gronovii*. Raw data were then processed using the ngs_backbone v.1.1.0 pipeline (Blanca *et al.*, 2011). After trimming, filtering, and quality assessment, Mira (version 3.2.0) (Chevreux *et al.*, 1999) was used to assemble the reads into contigs. The applied job options were ‘denovo, est’. The reliability of the assemblies was confirmed with an independent assembly strategy using the SeqClean Software for cleaning and the TGICL pipeline for the assembly (DFCI Gene Indices Software Tools, ftp://occams.dfci.harvard.edu/pub/bio/tgi/software/) (data not shown). Sequences were filtered using the Rfam (Griffiths-Jones *et al.*, 2005) and the SILVA (Quast *et al.*, 2013) databases. This Transcriptome Shotgun Assembly project has been deposited at DDBJ/EMBL/GenBank under the accession numbers GDKE00000000 (*C. gronovii*) and GDKD00000000 (*C. reflexa*). The versions described herein are the first versions, GDKE01000000 (*C. gronovii*) and GDKD01000000 (*C. reflexa*).

### *Analysis of* de novo *transcriptomes and SSH clones*

For functional annotation, the transcriptome contigs were analysed by sequence-based and domain-based alignments. Sequence-based alignments were performed with BLAST (blastx) (Altschul *et al.*, 1990) against the non-redundant (nr) protein database at NCBI (http://www.ncbi.nlm.nih.gov/protein) and the UniProt protein databases Swiss-Prot and UniRef90 (http://www.uniprot.org/). The E-value thresholds were set to 1e-15. Conserved protein domains were searched by using HMMER tools (HMMER 3.1, http://hmmer.janelia.org/) with the PFAM database (PFAM 27.0; Finn *et al.*, 2014). The online tool Mercator (Lohse *et al.*, 2014) was used to map all contigs to functional modules defined by the plant-specific ontology and pathway tool MapMan (Usadel *et al.*, 2005).

Sequences of the SSH clones were trimmed by deleting vector backbone sequences (pGEM-T Easy) and poly(A) tails before comparison with the *C. reflexa* transcriptome using BLAST (blastn; E-value: 1e-99). The sequences which did not give a match with any of the contigs in the *C. reflexa* reference collection were aligned in Geneious Pro 5.6.6 (Biomatters Ltd, Auckland, New Zealand) with ClustalW (Li, 2003). Functional annotation was again conducted with Mercator. The minimum BLAST bit score was set to 50.

### Phylogenetic analysis of XTHs

A rooted Neighbor–Joining tree was generated with PhyML on a ClustalW protein alignment of all *Arabidopsis thaliana* XTHs identified in the UniProt database (UniProt-Consortium, 2015), the two *C. reflexa* XTHs (Cr-XTH-1 and Cr-XTH-2) and a previously identified XTH, LeXTH1 (alias XTH1_Sly) from tomato, *Solanum lycopersicum* (Albert *et al.*, 2004). *Solanum lycopersicum* Expansin A23 served as the root. The Jones–Taylor–Thornton (JTT) model was used for amino acid substitutions, and the phylogram was optimized for substitution rates. One thousand bootstrap replications were conducted. All calculations were performed with Geneious Pro 5.6.6 (Biomatters Ltd).

### Reverse transcription quantitative real-time PCR (RT-qPCR)

SuperScript II Reverse Transcriptase (Invitrogen) and anchored oligo(dT)_18_ primers were used to synthesize cDNA from 1 µg of DNase-treated total RNA. Controls without reverse transcriptase were carried out for each target gene in order to verify the complete absence of contaminating DNA. Quantitative real-time PCR was performed in technical duplicates using the SsoFast EvaGreen Supermix (Bio-Rad, Oslo, Norway) according to the manufacturer’s specifications. Thermal cycling and fluorescence detection was carried out using a CFX96 Real-Time PCR Detection System (Bio-Rad) with the following cycling conditions: 95 °C for 30s followed by 40 cycles of 95 °C for 5s and 61 °C for 5s. After 40 cycles, melt curves were recorded by stepwise heating from 65 °C to 95 °C. The amplification efficiencies were taken into account when calculating the relative abundances of each target gene (Pfaffl, 2001). Relative abundances of *C. reflexa* (*Cr*)*-ACTIN* and *Cr-SF2* were used to normalize the expression levels between *C. reflexa* samples. *Cuscuta gronovii* (*Cg*)-*ACTIN* and *Cg-SF2* were used as reference genes for *C. gronovii*. In samples where a target transcript could not be detected in any of the technical duplicates [i.e. no Cq (quantification cycle) value], a Cq value was assigned by adding one cycle to the highest Cq in that run. Data were analysed with the CFX Manager Software 2.0 (Bio-Rad). Gene-specific primer sequences with their respective amplicon sizes and PCR efficiencies are listed in Supplementary Table S1 available at *JXB* online. Amplicon melt curve analyses and size separation on agarose gels are presented in Supplementary Figs S1 and S2, respectively.

### Immunolocalization of XTHs

Cross-sections (70 µm) of haustoria 3 d after FR light induction were prepared using a Leica VT1000E vibratome (Leica Biosystems, Nussloch GmbH, Nussloch, Germany). Free binding sites were blocked for 30min with 5% (w/v) non-fat milk powder in standard phosphate-buffered saline buffer (1× PBS) (blocking buffer). After washing with PBS, 1:20 dilutions of polyclonal anti-XTH rabbit IgGs or IgGs from the pre-immune serum (both provided by Dr E. Labrador and her group at the University of Salamanca, Spain) in blocking buffer were applied to the sections for 2h followed by three 5min washes with PBS. The sections were then incubated for 1h in the dark with Alexa Fluor 555 Goat Anti-Rabbit IgG (Life Technologies, Carlsbad, CA, USA) (1:200 in blocking buffer) and subsequently washed with PBS. Labelled sections were stained with toluidine blue O in order to quench autofluorescence. Micrographs were taken with a SteREO Lumar V12 equipped with an AxioCam MRc5 camera and the Lumar 43 filter set (all from Carl Zeiss, Jena, Germany).

### XET action assays

XET test papers were prepared as described by Fry (1997). A piece of Whatman No. 1 filter paper was passed over the surface of 1% (w/v) Tamarind xyloglucan (Megazyme, Ireland) dissolved in 0.5% (w/v) chlorobutanol and left to dry. The dry xyloglucan-coated paper was dipped in 5 µM sulphorhodamine-labelled xyloglucan oligosaccharides (XyGO-SR) dissolved in 75% (v/v) acetone and dried before use. XyGO-SR were prepared by conjugation of xyloglucan oligosaccharides with sulphorhodamine carried out as described by Kosik and Farkas (2008). Xyloglucan-coated papers without XyGO-SR were used as control papers. Tissue prints were made by placing hand-cut 0.5–1.0mm thick pieces of *Cuscuta* cross-sections on XET test papers or control papers soaked in 50mM Na-acetate, 300mM NaCl (pH 5.5) followed by incubation between two sheets of acetate for 1h. The background on printed papers was removed by washing in water:ethanol:formic acid (1:1:1) for 2h with gentle agitation followed by rinsing in distilled water. Fluorescence micrographs were taken of dried de-stained papers using a SteREO Lumar V12 equipped as described above.

## Results

### Identification of haustorium-specific gene transcripts

**Fig. 2.**
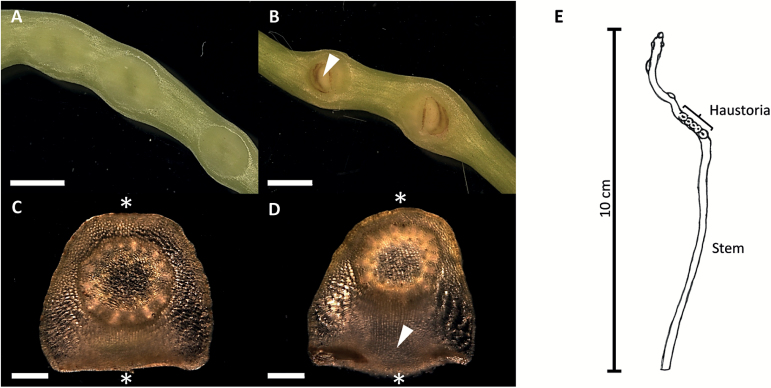
Haustorium development of FR light-induced *C. reflexa* shoots. (A and C) Initial stages of haustoriogenesis were visible 3 d after treatment with FR light. (B and D) After 6 d, the haustorial body (arrowheads in B and D) could be discerned. Asterisks indicate sites of contact with the plastic surface of the Petri dish. (E) Schematic overview of FR light-induced *C. reflexa* shoots indicating the young haustoria and stem areas used for SSH. Cross-sections were made with respect to the orientation of the parasite shoot axis. Scale bars are 2000 µm (A and B) and 500 µm (C and D).

Tada *et al.* (1996) reported that the formation of haustoria in *C. japonica* can be induced through the synergistic effect of FR light and tactile stimuli without the presence of a host plant. In the present study, the same signals were applied to activate haustorium differentiation in *C. reflexa* and *C. gronovii*. While host plant infection by *Cuscuta* progressed with varying speed, the development of FR light-induced haustoria proceeded in a very predictable manner: 3 d after treatment with FR light the parasite stem was noticeably swollen at one side, and after 6 d the haustorial body could be discerned (shown for *C. reflexa* in Fig. 2). Cross-sections of these young haustorial stages (Fig. 2C, D) resembled those during early host infection (Fig. 1F). The predictability of haustorial onset is, of course, a distinctive advantage when searching for developmentally regulated genes in the *Cuscuta* haustorium. Moreover, the intimate connection between parasite and infected host, and the translocation of transcripts from host to parasite through the haustorium (Leblanc *et al.*, 2012; Kim *et al.*, 2014), makes it difficult to avoid contamination of the RNA from haustorial infection sites with host-encoded transcripts. The concentration of host mRNA in *Cuscuta* is generally increased towards the host–parasite interface (Leblanc *et al.*, 2013), which would lead to an enrichment of host transcripts during the SSH procedure and consequently mask the differences in parasitic gene expression (Diatchenko *et al.*, 1996). Therefore, haustoria and stem tissue from FR light-induced shoot tips of *C. reflexa* were used for the generation of differentially expressed SSH libraries (Fig. 2E).

**Fig. 3.**
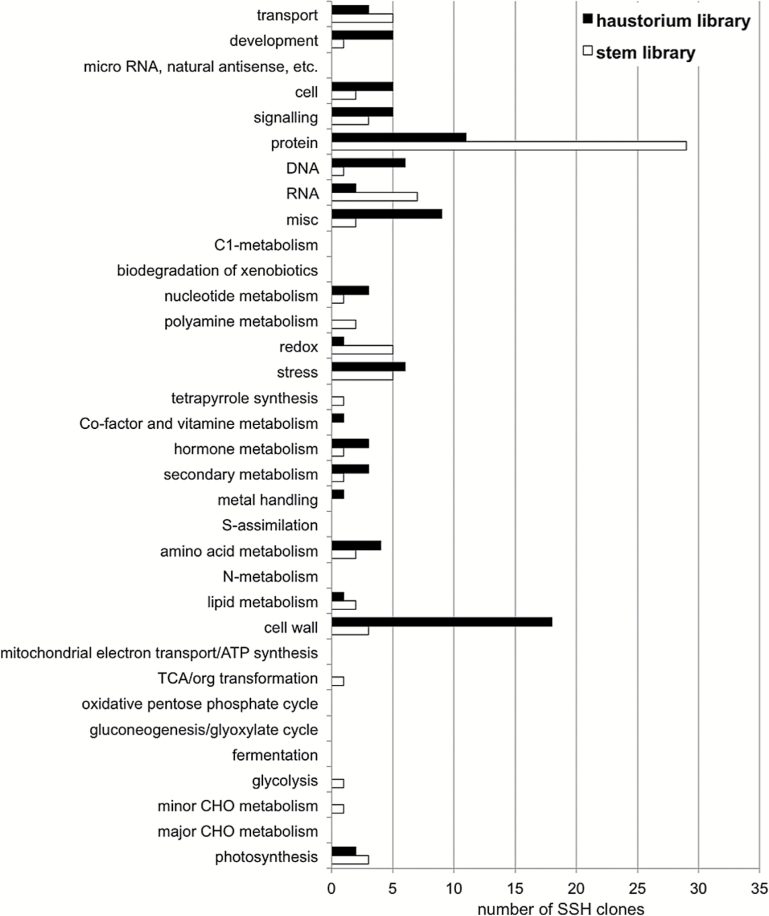
MapMan binning of *C. reflexa* SSH clones. The clones were mapped and functionally classified using the online tool Mercator. The minimum BLAST bit score was set to 50.

The SSH procedure is directional, only enriching transcripts that are present in higher abundance in one sample (tester) compared with another (driver). In this study, the SSH was carried out in both directions, leading to the construction of two libraries: the haustorium library enriched for sequences that are more abundant in young (3-day-old) haustoria than in the stem; and the reciprocal stem library enriched for sequences with higher expression in the stem tissue. Clones from both libraries were randomly picked and sequenced, yielding 182 and 179 readable sequences from the stem- and haustorium-specific libraries, respectively, which were subjected to an analysis by the functional annotation tool Mercator (Lohse *et al.*, 2014). Mercator combines sequence similarity searches to a variety of plant genomes as well as information derived from InterProScan, KOG, and cdd searches, and assigns them to so-called bins representing different functional categories. Seventy-nine stem-specific sequences and 89 haustorium-specific sequences could be confidently assigned to a MapMan category. The quantitative distribution of assigned functions showed that particularly transcripts associated with the bins ‘cell wall’, ‘DNA’, ‘development’, and ‘misc’ were frequent in the haustorium-specific library but not in the stem-specific library (Fig. 3). The stem library, on the other hand, contained more transcripts for genes associated with the bins ‘protein’, ‘RNA’, and ‘redox’.

### De novo *sequencing of transcriptomes of two* Cuscuta *species*

To create a ‘baseline’ for the relative sizes of MapMan categories in *Cuscuta*, the representation of MapMan bins in entire transcriptomes was investigated. For that purpose, 454-based *de novo* sequencing of the mRNAs from pooled vegetative tissue of *C. reflexa* and a second species, *C. gronovii*, was performed. These reference transcriptomes were also expected to improve the sequence information for the transcripts of interest obtained by the SSH screen. One and one-eighth 454 runs (see the Materials and methods) yielded just under 1×10^6^ raw reads per species, which resulted in a total of 42 103 and 31 685 contigs for *C. reflexa* and *C. gronovii*, respectively (Table 1). Both the average and largest contig sizes were only marginally larger in *C. reflexa* than in *C. gronovii*. To predict the function of the *Cuscuta* contigs, their sequences were compared with different databases. The NCBI nr database, the UniProt Swiss-Prot and the UniRef90 databases individually yielded hits with up to 96% of the sequences for both species (Table 2). All approaches combined (i.e. nr, Swiss-Prot, and PFAM) allowed the annotation of 98% of the contigs of *C. reflexa* and of *C. gronovii* (Table 2).

**Table 1.** Summary of 454 sequencing data and contig assembly

	*C. gronovii*	*C. reflexa*
**Number of raw sequences**	914 135	945 254
**Number of contigs**	31 685	42 103
**Mean contig size**	800.4 bp	843.9 bp
**Largest contig**	5 965 bp	6 735 bp
**N25**	1 318 bp	1 391 bp
**N50**	867 bp	906 bp
**N75**	617 bp	642 bp

**Table 2.** Annotation of *Cuscuta* contigs The number of contigs with hits to annotated sequences in public databases (blastx thresholds E-value: 1e-15) and with no annotation are shown (percentages in parentheses).

	nr (NCBI)	SwissProt (UniProt)	UniRef90 (UniProt)	PFAM 27.0 (EMBL-EBI)	No annotation
***C. gronovii***	29 052 (92%)	30 105 (95%)	30 347 (96%)	13 780 (43%)	555 (2%)
***C. reflexa***	38 944 (92%)	39 465 (94%)	39 927 (95%)	18 648 (44%)	984 (2%)

**Fig. 4.**
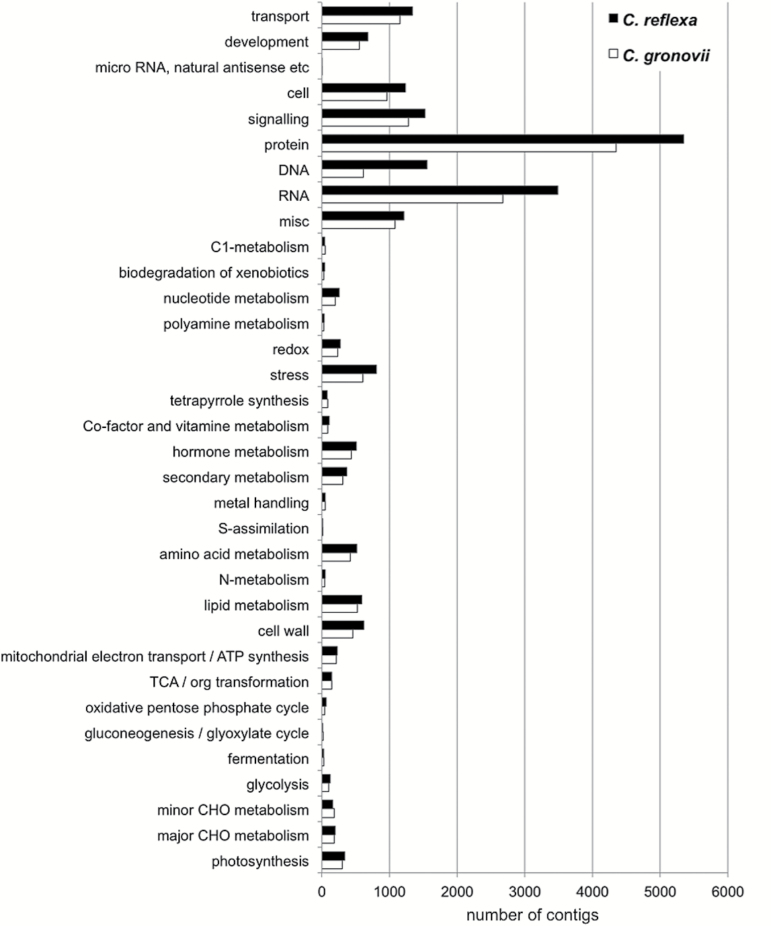
MapMan binning of *Cuscuta* transcriptome contigs. The contigs were mapped and functionally classified using the online tool Mercator. The minimum BLAST bit score was set to 50.

Using Mercator (Lohse *et al.*, 2014), 52% of the *C. reflexa* contigs and 55% of the *C. gronovii* contigs could be assigned to bins. The distribution chart in Fig. 4 shows that most cellular functions are covered by the contig collections of both *Cuscuta* species and that the relative distribution of contigs to most of the bins is overall similar. Cell wall-related transcripts were not over-represented in the total transcriptomes (Fig. 4), substantiating the suspicion that their abundance in the haustorium-specific SSH cDNA library (Fig. 3) reflects elevated cell wall remodelling activities in this tissue.

**Fig. 5.**
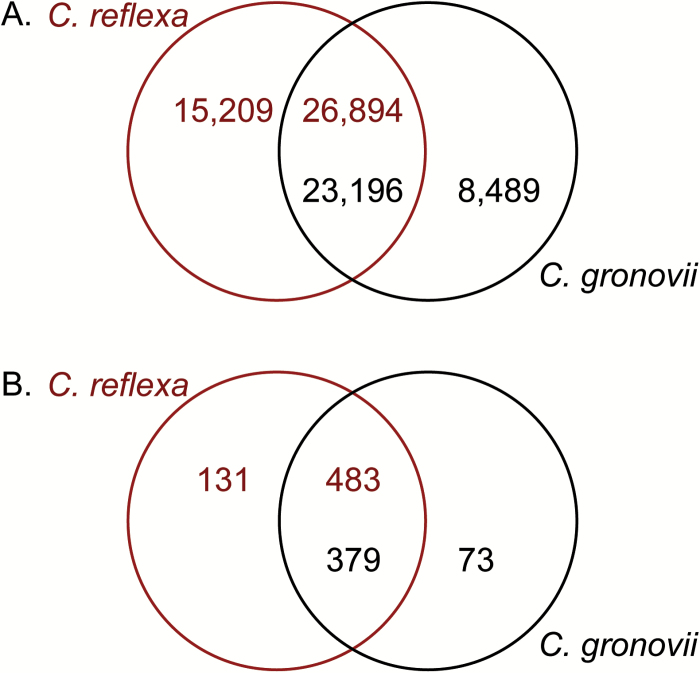
Contig overlap between the two *Cuscuta* species (A) for all contigs and (B) for contigs related to cell wall functions. The Venn diagrams show how many contigs in the *C. reflexa* (top row) and *C. gronovii* (bottom row) transcriptomes have homologues in the other species (blastn threshold E-value: 1e-10). (This figure is available in colour at *JXB* online.)

A direct sequence homology comparison between the two *Cuscuta* species showed that 73% (23 196) of the *C. gronovii* contigs had homologues in the *C. reflexa* transcriptome, while 64% (26 894) of the *C. reflexa* transcripts were also retrieved in *C. gronovii* (Fig. 5A). The difference in the percentages is at least in part due to the lower number of contigs assembled for *C. gronovii*. For contigs belonging to the cell wall-related bin, congruencies were even a bit higher. Of the 452 (*C. gronovii*) and 614 (*C. reflexa*) contigs that were sorted to this bin, 379 (84%) and 483 (79%), respectively, were also found in the other *Cuscuta* species (Fig. 5B).

### Validation of differential expression by RT-qPCR

The reference transcriptomes described above provided information on complete or almost complete cDNA sequences for most of the partial cDNA clones that were recovered from the SSH libraries. With this additional sequence information, RT-qPCR primers for validation of the differential transcript accumulation indicated by the SSH were generated. To this end, the relative expression levels of 10 genes from the haustorium library and two genes from the stem library putatively related to cell wall functions were quantified in the RNA samples used for the SSH by RT-qPCR. All genes displayed fold changes that reflected the differential expression suggested by the SSH; the two stem-specific genes (*Cr-GH5* and *Cr-GT20*) were less expressed in young haustoria compared with the stem, whereas the transcript abundances of the 10 haustorium-specific genes (*Cr-GH1*, *Cr-GH17*, *Cr-GH31*, *Cr-PAE*, *Cr-PX-1*, *Cr-PX-2*, *Cr-PX-3*, *Cr-RGP*, *Cr-XTH-1*, and *Cr-XTH-2*) were higher in the haustorial tissue (Table 3).

**Table 3.** Validation of SSH-identified differentially expressed cell wall-related genes by RT-qPCR Gene designations, the corresponding SSH library, and fold changes ±SD of technical duplicates are shown.

**SSH library**	**Gene**	**Fold change (H:S**)^***a***^
**Haustorium**	*Cr-GH1*	15±2.3
	*Cr-GH17*	2.2±0.54
	*Cr-GH31*	25±3.6
	*Cr-PAE*	11±2.8
	*Cr-PX-1*	1.9±0.33
	*Cr-PX-2*	781^*b*^
	*Cr-PX-3*	8.2±1.2
	*Cr-RGP*	13±1.9
	*Cr-XTH-1*	129±21
	*Cr-XTH-2*	195±29
**Stem**	*Cr-GH5*	–1.7±0.25
	*Cr-GT20*	–16±4.8

^*a*^ Values are means of technical duplicates in the normalized transcript abundances of the RNA sample used to generate the tester cDNA for the haustorium library (H) compared with the RNA sample used to generate the tester cDNA for the stem library (S). Reference genes: *Cr-ACTIN* and *Cr-SF2*.

^*b*^ No SD because target transcript could not be detected in the stem RNA sample.

### Expression mapping of differentially expressed genes

**Fig. 6.**
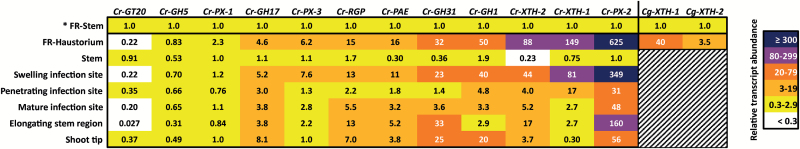
Expression of cell wall-related genes in tissues of *Cuscuta*. Transcript abundances in relation to the transcript abundances in **‘**FR-Stem**’** (* set to 1) are presented in a heat map with colours ranging from light (<0.3) to dark (≥300). All transcript abundances in *C. reflexa* and *C. gronovii* are normalized to the abundances of *Cr-ACTIN* and *Cr-SF2* transcripts, and the abundances of *Cg-ACTIN* and *Cg-SF2* transcripts, respectively. Values are the mean of three biological replicates. (This figure is available in colour at *JXB* online.)

In order to investigate if these tentative haustorium-associated genes were also expressed during host-induced haustoriogenesis and if their expression is maintained during later stages of haustorium development or not, the gene-specific transcript abundances were quantified in swelling, penetrating, and mature stages of *C. reflexa* infecting *P. zonale*. All genes displayed similar expression levels in the early swelling stage of the host infection to those in the FR light-induced haustoria (Fig. 6). The expression in the stems was also similar, indicating that FR light irradiation in itself had no severe effect on the expression of these genes. Interestingly, *Cr-RGP*, *Cr-PAE*, two glycoside hydrolase members (*Cr-GH31* and *Cr-GH1*), the two XTH genes (*Cr-XTH-1* and *Cr-XTH-2*), and the peroxidase genes *Cr-PX-2* and *Cr-PX-3* showed a considerable decrease in expression levels in the more advanced stages of haustorium development (Fig. 6). To examine further if these genes were expressed in young haustoria only as a result of the high rates of cell division and cell elongation in these organs, the expression levels were also quantified in growing shoot tips, where the apical meristem is located, and in the stem region just below the tip that displays high rates of elongation (see Supplementary Fig. S3 at *JXB* online). While some genes indeed displayed an expression behaviour that suggests a general association with either cell division, cell elongation, or both (e.g. *Cr-GH17*, *Cr-RGP*, and *Cr-GH31*), the two *Cr-XTH* genes and *Cr-PX-2* showed a more differentiated expression pattern with a clear increase in young haustoria (Fig. 6). These three genes displayed the highest differential expression levels, with changes of ≥80-fold in both our experiments (Table 3; Fig. 6). Moreover, the closest homologues to *Cr-XTH-1* and *Cr-XTH-2* in *C. gronovii*, *Cg-XTH-1* and *Cg-XTH-2*, were both more highly expressed in young FR light-induced haustoria than in stems of this species (Fig. 6). The numerical values of all gene expression levels are presented in Supplementary Tables S2 and S3.

**Fig. 7.**
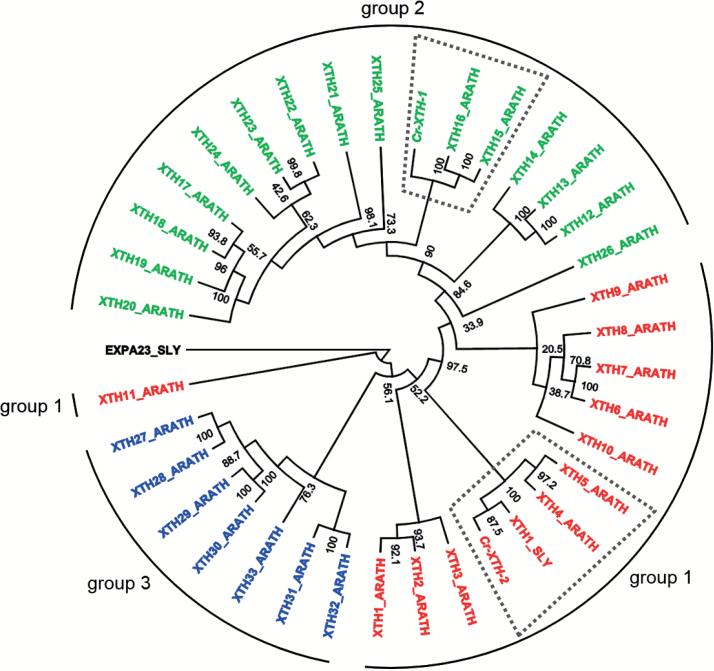
Neighbor–Joining tree showing the relationship between *C. reflexa* XTH-1 and XTH-2 and *Solanum lycopersicum* XTH1 (XTH1_SLY, formerly LeXTH1) based on their phylogenetic placement within the *Arabidopsis thaliana* XTH gene family tree. The tree was rooted with *S. lycopersicum* Expansin A23 (EXPA23_SLY). The phylogram was optimized for substitution rates. Numbers next to the nodes represent bootstrap proportion values from 1000 replications. Designation of the subgroups 1**–**3 of the XTH protein family of *A. thaliana* are adopted from Rose *et al.* (2002). Clusters that include Cr-XTH-1, Cr-XTH-2, and XTH1_SLY are accentuated by a surrounding broken line. (This figure is available in colour at *JXB* online.)

Interestingly, the increased expression of a tomato gene encoding an XTH, *Lycopersicum esculentum LeXTH1* (here referred to as *S. lycopersicum XTH1_SLY*), has been described as a possible defence reaction of an incompatible tomato plant being attacked by *C. reflexa* potentially by tightening the cell walls (Albert *et al.*, 2004). A comparison of the XTH1_SLY protein sequence and the *Cuscuta* XTHs showed that the tomato protein is very similar to Cr-XTH-2 and that both cluster with the *A. thaliana* XTH4 and XTH5 proteins in group 1 (Rose *et al.*, 2002) (Fig. 7). In contrast, Cr-XTH-1 clusters with *A. thaliana* XTH15 and XTH16 within group 2 of this protein family.

### Presence and distribution of XTH proteins and of XET action

**Fig. 8.**
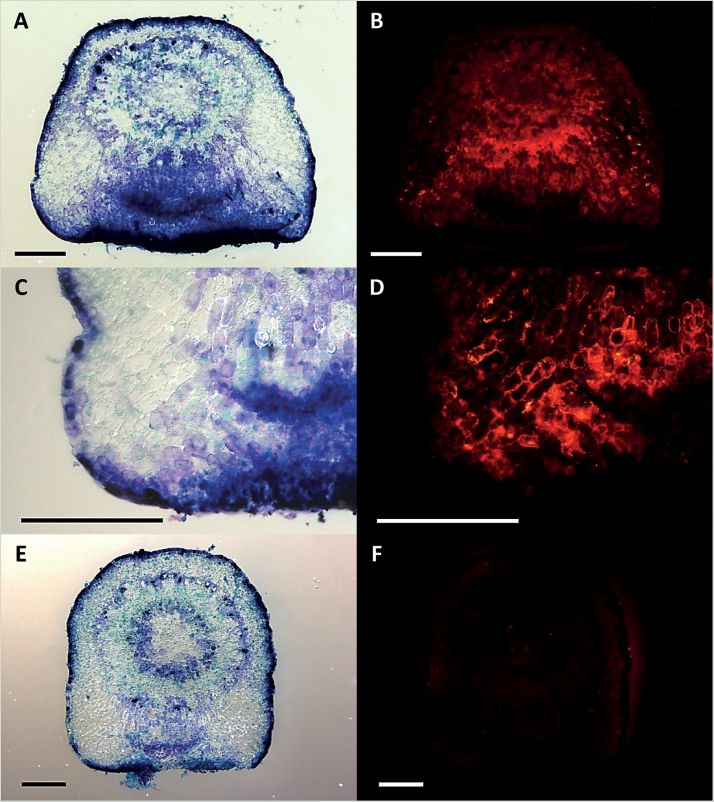
Localization of XTHs in young FR light-induced haustoria. (A and C) Bright field and (B and D) fluorescence micrographs of toluidine blue O-stained and XTH-labelled cross-sections of haustoria 3 d after treatment with FR light. (E) Bright field and (F) fluorescence micrographs of control with pre-immune IgGs. Scale bars are 500 µm.

**Fig. 9.**
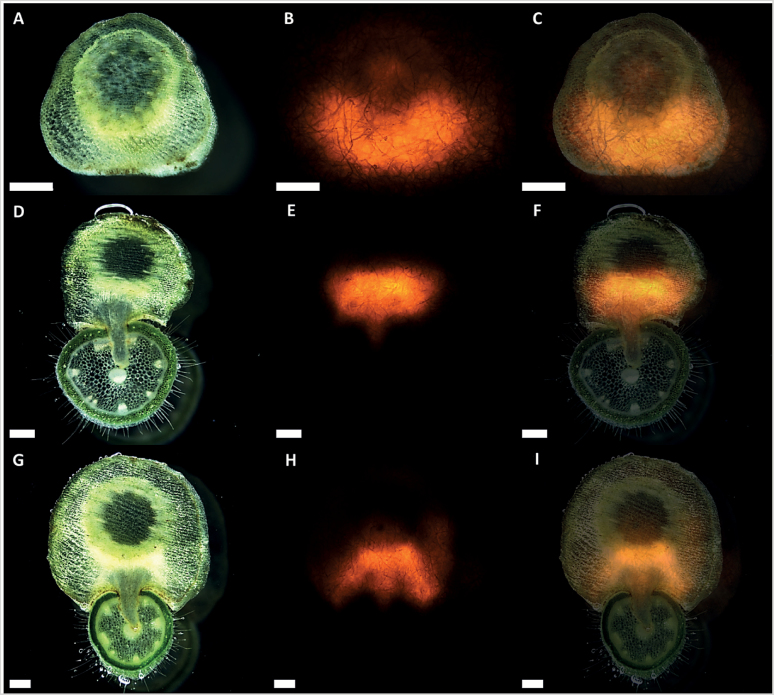
XET action during infection of *P. zonale* by *C. reflexa*. Cross-sections of (A) swelling, (D) penetrating, and (G) mature infection stages tissue printed on XET test paper. (B, E, and H) Fluorescence micrographs showing XET action in the printed tissues in (A), (D), and (G), respectively. Merged pictures of (A–B), (D–E), and (G–H) are presented in (C), (F), and (I), respectively. Scale bars are 500 µm. Visible fibres are integral to test papers.

To approach the putative functionality of XTHs in *Cuscuta* haustorium development, an XTH-specific antibody (Jimenez *et al.*, 2006) was used to label the proteins in cross-sections of FR light-induced haustoria. Fluorescence micrographs indicated that these xyloglucan-modifying enzymes are located in the cell walls of elongating cells in the areas flanking the haustorial initiation centre (Fig. 8); that is, in the part that is responsible for the swelling of the stem during attachment to a host plant. Furthermore, the xyloglucan endotransglucosylation action of XTHs was analysed in cross-sections of young FR light-induced haustoria as well as swelling, penetrating, and mature infection sites of *C. reflexa* infecting *P. zonale* by *in situ* XET action assays. Xyloglucan endotransglucosylation was detected in the parasite throughout haustorium development, with a clear bias towards the side facing the host plant (Fig. 9). Weaker XET action was also detected in the endophytic part of the haustorium. In contrast, the host plant did not display XTH-specific action. Tissue printing on control papers did not produce any fluorescent signal (data not shown). The detected XET action in the swelling areas of FR light-induced haustoria correlated with the immunolocalization patterns observed for these proteins (Supplementary Fig. S4 at *JXB* online). In contrast, enzyme extracts from *Cuscuta* haustoria had no detectable hydrolytic activity towards Tamarind xyloglucan (Supplementary Fig. S5).

## Discussion

Most existing studies on the specific gene expression changes unfolding during the interaction between species of *Cuscuta* and their hosts have focused on genes expressed by the host upon parasitization. Differentially expressed host genes representing various functions, including general plant pathogen defence responses and cell wall metabolism, were identified by differential display or SSH techniques (Borsics and Lados, 2002; Albert *et al.*, 2004; Li *et al.*, 2009). A study on differential gene expression in *Cuscuta* has identified a cysteine protease, Cuscutain (Bleischwitz *et al.*, 2010), but with the exception of this, corresponding studies are very scarce. Two aspects potentially aggravate the identification of *bona fide* differentially expressed genes during *Cuscuta* haustorium development: the uptake of host RNAs (Kim and Westwood, 2015) and the lack of a genome sequence from any *Cuscuta* species. In this study, these risks were mitigated by inducing haustoriogenesis in the absence of a host plant and by generating reference transcriptomes from two *Cuscuta* species using tissue that was not in contact with the host.

FR light in the range of 740nm together with physical contact between the parasite and a surface (normally the host) were described to be effective inducers of haustorium development in the absence of a host plant (Tada *et al.*, 1996). The reversal of the inductive effect by red light (660nm) further suggested the involvement of phytochrome in haustorium differentiation (Furuhashi *et al.*, 1997). FR light also triggers positive phototropism in seedlings of *Cuscuta*, prompting speculations that this fraction of the visible light that passes the green foliage provides an indication to the parasite that hosts are present (Orr *et al.*, 1996). Morphologically, the early stages of haustorium development on a host plant and after FR light induction are similar (Figs 1, 2). Moreover, the present study demonstrated that all differentially expressed candidate genes, whose expression levels were quantified by RT-qPCR, displayed the same tendencies in the first stage of FR light- and host-induced haustoriogenesis (Fig. 6). This suggests that the physical stimuli sufficed to induce the transcriptional reprogramming and the ensuing tissue differentiation. The initial swelling stage during which the parasite attaches itself to the surface is followed by the protrusion of the discernible haustorium at ~6 d after FR light treatment (Fig. 2). Also at this advanced stage, gene-specific transcript abundances were comparable with those in penetrating stages of host plant infection (Supplementary Table S3 at *JXB* online), substantiating the conclusion that in these stages of haustorium development, changes in the transcriptional pattern do not depend on any host-derived signals. Ultimately, however, the plastic Petri dish hinders further growth of the haustorium, while in a host the differentiation of feeding hyphae commences. This last step that leads to an establishment of a physiological connection appears to be dependent on specific signals from the tissue that is invaded (Christensen *et al.*, 2003; Vaughn, 2006) and can therefore not be easily mimicked in a host-free system.

An analysis of the 394 SSH clones whose inserts were sequenced (198 and 196, respectively, for each library), already showed a clear bias towards different functional categories represented by the transcripts in each of the two SSH libraries. The most conspicuous bias was that towards cell wall-related functions in the haustorium-specific library (Figs 3, 4). This finding conforms well with recent reports of changes to the cell wall composition during host-invasive growth of *Cuscuta* (Johnsen *et al.*, 2015; Striberny and Krause, 2015). We therefore did not conduct more in-depth sequencing of the differentially expressed cDNA libraries and instead proceeded to profile the expression of cell wall-related genes during haustorium development and in comparison with other *Cuscuta* tissues exhibiting rapid growth. In haustoria, cell division and cell elongation are most pronounced in the shoot tips and a few centimetres below the tips, respectively (Supplementary Figure S3 at *JXB* online). Quantitative expression analysis in these parts of the parasite can thus reveal whether the identified genes are associated with growth in general or whether they are specific to haustoriogenesis.

*Cr-GH17* and *Cr-GH31* are glycoside hydrolases that displayed expression levels in elongating stem regions and/or shoot tips of *C. reflexa* that were comparable with the expression levels in the young haustorial tissues (Fig. 6). The products of these genes are therefore not unique to the haustorium, but rather are characteristic for growing tissues of the parasite. The GHs are a large and widespread group of enzymes characterized by their ability to hydrolyse glycosidic bonds, thus facilitating breakdown of carbohydrates. The majority of GHs in plants are involved in cell wall metabolism (Frankova and Fry, 2013).

A third gene, *Cr-RGP*, which encodes a reversibly glycosylated polypeptide, also showed elevated expression levels in non-haustorial growing tissues of the parasite. RGPs are a family of proteins suggested to be involved in the synthesis of plant cell wall polysaccharides (Dhugga *et al.*, 1997), but whose exact function remains elusive. Based on sequence similarity, *Cr-RGP* belongs to class 1 RGPs (Langeveld *et al.*, 2002), which are tentatively associated with plasmodesmata (Sagi *et al.*, 2005).

Several studies connect pectins and its modifiers to host plant infection by *Cuscuta* (Nagar *et al.*, 1984; Srivastava *et al.*, 1994; Bar Nun and Mayer, 1999; Vaughn, 2002, 2003, 2006; Johnsen *et al.*, 2015). In the present study, the transcript abundance of a pectin acetylesterase, *Cr-PAE*, was found to be higher in young haustoria than in all other tested tissues of *Cuscuta* (Fig. 6), making it a potential marker gene for haustorium initiation and corroborating the role of pectin modification during the infection process.

*Cr-PX-2* and *Cr-PX-3* transcripts were also more abundant in young haustoria than in any of the other tissues (Fig. 6). Peroxidases are a large group of enzymes that catalyse the oxidation of a number of substrates (Battistuzzi *et al.*, 2010). In plants, PXs are associated with cell wall metabolism (Duroux and Welinder, 2003; Veitch, 2004) and with the defence against plant pathogens (Lamb and Dixon, 1997; Lopez-Curto *et al.*, 2006) so their relative abundance may indicate a ‘state of alert’ in the haustorial cells of *Cuscuta*.

Expression differences that were similarly large or even larger than those of the peroxidase genes and likewise specific to the young haustoria of *C. reflexa* were observed for *Cr-XTH-1* and *Cr-XTH-2.* XTHs are assigned roles in loosening of the plant cell wall by restructuring xyloglucans, allowing turgor-driven expansive cell growth (Rose *et al.*, 2002; Van Sandt *et al.*, 2007). Xyloglucans are the most abundant hemicelluloses in primary cell walls of dicotyledonous plants (Scheller and Ulvskov, 2010) so that all parasitic plants can be expected to display xyloglucan-modifying activities. The differential expression of closely related XTH genes in FR light-induced haustoria of *C. gronovii*, which belongs to a subgenus of *Cuscuta* different than *C. reflexa*, substantiates the assumption (Fig. 6). Also, Ranjan *et al.* (2014) found seven *XTH* genes in *C. pentagona* that were more highly expressed at the pre-haustorial stage compared with seedlings and stems, suggesting that the early expression of these genes is in fact part of a common developmental pattern in most, if not all, *Cuscuta* species.

Recent epitope deletion assays (Vidal-Melgosa *et al.*, 2015) showed that specifically the group of xyloglucans containing the XXXG-motif is a target of enzymatic activity in haustoria and in the infected host (Johnsen *et al.*, 2015). In the present study, these previous investigations on xyloglucan-modifying enzymes were extended by immunolocalization studies and XET action assays using tissue prints on XET test papers. Anti-XTH labelling of 3-day-old FR light-induced haustoria confined the xyloglucan-modifying enzymes to the cell walls of elongating cells in the swelling areas of young haustoria (Fig. 8), indicating that restructuring of this hemicellulose is taking place during the initial stage of haustoriogenesis. *In vitro* activity assays using crude extracts suggested that the xyloglucan restructuring activity was not predominantly hydrolytic (see Supplementary Fig. S5 at *JXB* online). Correspondingly, *in situ* XET action assays clearly revealed xyloglucan endotransglucosylation in the swelling area of FR light-induced haustoria (Supplementary Fig. S4) and in the comparable area of haustoria that faces the host plant during infection (Fig. 9). Vaughn (2006) reported reduced cellulose and xyloglucan levels in the cell walls of phloic hyphae during the parasitization of *Impatiens* by *C. pentagona* and proposed that this loosening of the cell wall facilitates apoplastic transfer of sugars into the parasite. A reduction in xyloglucan in the neighbouring phloem cells of the host was also observed in the same study. Some XET action was detected in the endophytic part of the haustorium in the present study (Fig. 9). Since *Cr-XTH-1* and *Cr-XTH-2* expression levels drop in the more advanced stages of haustoriogenesis, a full investigation of the expression patterns of all other *XTH* gene candidates that have been identified in the reference transcriptomes of both *Cuscuta* species will be necessary to reveal which of the genes take over the hypothetical task of modifying host xyloglucans.

Although the exact function of neither Cr-XTH-2 nor XTH1_SLY is known, their intriguing similarity (Fig. 7) gives rise to a bold train of thought where the infective mechanisms in parasites might not have evolved *de novo* but, rather, through re-purposing of defence pathways that existed already in their non-parasitic ancestors. A similar scenario was suggested as an explanation for similarities in the mutual recognition systems by pathogens and hosts (Vasta, 2012). *XTH1_SLY* is expressed very strongly in expanding tomato fruits (Matas *et al.*, 2011) (see also Tomato eFP Browser, http://bar.utoronto.ca/efp_tomato/cgi-bin/efpWeb.cgi), suggesting a role during fruit ripening. While this can be best reconciled with a molecular function in cell wall loosening, XTHs were also shown to have the opposite effect and can tighten cell walls (Maris *et al.*, 2009). In fact, the latter process would be expected to exist in the parasite to protect its walls from its own hydrolytic enzyme cocktail, but such details of the complex array of actions and reactions between *Cuscuta* and its hosts need to be addressed in future work.

Our observation that the expression of cell wall-related genes in *Cuscuta* was up-regulated at the onset of haustorium development is in agreement with other recent reports on gene expression in parasitic plants (Ranjan *et al.*, 2014; Yang *et al.*, 2014), which indicates that changes to cell walls are essential to the formation of the infection organ. Furthermore, the site-specific action of XTHs during host plant infection points towards the relevance of xyloglucan modification in host–*Cuscuta* interactions. Consequently, cell wall genes and *XTH* genes in particular might prove effective gene silencing targets for the control of *Cuscuta* in agriculture, and, as such, efforts to unravel the role of xyloglucan and its modifiers in haustorium development should be continued.

## Supplementary data

Supplementary data are available at *JXB* online.

Supplementary Table S1. Sequences of gene-specific primers with respective amplicon sizes and PCR efficiencies.

Supplementary Table S2. Gene expression levels in single biological replicates.

Supplementary Table S3. Mean gene expression levels of biological triplicates.

Supplementary Fig. S1. Melt peaks of qPCR amplicons.

Supplementary Fig. S2. Size separation of qPCR amplicons on agarose gel.

Supplementary Fig.S3. Stem elongation in the region just below the apical shoot tip.

Supplementary Fig. S4. XET action in FR light-induced haustoria.

Supplementary Fig. S5. Hydrolytic activity of a haustorial enzyme extract from *C. reflexa* towards xyloglucan.

Supplementary Data

## Abbreviations:

Cg: *Cuscuta gronovii*
Cq: quantification cycle
Cr: *Cuscuta reflexa*
FR: far-red
GH: glycoside hydrolase
GT: glycosyltransferase
PAE: pectin acetylesterase
PX: peroxidase
RGP: reversibly glycosylated polypeptide
RT-qPCR: reverse transcription quantitative real-time PCR
SF2: pre-mRNA splicing factor 2
SSH: suppression subtractive hybridization
XET: xyloglucan endotransglucosylation
XTH: xyloglucan endotransglucosylase/hydrolase.
