# Supplementary material for: Getting ready for host invasion: elevated expression and action of xyloglucan endotransglucosylases/hydrolases in developing haustoria of the holoparasitic angiosperm Cuscuta
Source: J Exp Bot. 2015 Nov 11;67(3):695–708. doi: 10.1093/jxb/erv482 (PMC4737069; doi:10.1093/jxb/erv482)
Supplement: Supplementary Data [file supp_67_3_695__index.html]

Getting ready for host invasion: elevated expression and action of xyloglucan endotransglucosylases/hydrolases in developing haustoria of the holoparasitic angiosperm Cuscuta — Getting ready for host invasion: elevated expression and action of xyloglucan endotransglucosylases/hydrolases in developing haustoria of the holoparasitic angiosperm Cuscuta — Supplementary Data 

# Getting ready for host invasion: elevated expression and action of xyloglucan endotransglucosylases/hydrolases in developing haustoria of the holoparasitic angiosperm *Cuscuta*

## Supplementary Data

Data files

- Supplementary\_Tables\_S1\_Figures\_S1\_S5.pdf - Supplementary Data
- Supplementary\_Tables\_S2\_S3.xlsx - Supplementary Data
