# Supplementary material for: Getting ready for host invasion: elevated expression and action of xyloglucan endotransglucosylases/hydrolases in developing haustoria of the holoparasitic angiosperm Cuscuta
Source: J Exp Bot. 2015 Nov 11;67(3):695–708. doi: 10.1093/jxb/erv482 (PMC4737069; doi:10.1093/jxb/erv482)
Supplement: Supplementary Data [file supp_erv482_Supplementary_Tables_S1_Figures_S1_S5.pdf]

Supplementary data for

"Getting ready for host invasion: elevated expression and action of xyloglucan endotransglucosylases/hydrolases in developing haustoria of the holoparasitic angiosperm *Cuscuta*"

Stian Olsen, Bernd Striberny, Julien Hollmann, Rainer Schwacke, Zoë Popper and Kirsten Krause

Supplementary Table S1. Sequences of gene-specific primers with respective amplicon sizes and PCR efficiencies.

Gene	Forward primer (5`→3`)	Reverse primer (5`→3`)	Amplicon size	Efficiency	R^2
Cr-ACTIN	ATGGAAGCTGCTGGAATCCAC	TTGCTCATACGGTCAGCGATG	140 bp	96.3 %	0.999
Cr-SF2	CGAGGATTTGTTTTACAAGTATGG	CGACCACGAATAGCGTCTTCC	126 bp	102.3 %	0.998
Cr-GH1	TGGTATGGGGTATGCCTCTC	AACCAGCCAAACACGAAATC	82 bp	105.7 %	0.991
Cr-GH31	TCGGTTCCACGACCTTTTAC	TTGCAGTCCAAACAGCAGAC	147 bp	105.9 %	0.996
Cr-GH17	CAAGTTCTTTTGTCCCACCAA	CGAACACCCTTTTCCACTACA	144 bp	99.6 %	0.990
Cr-XTH-1	CACTTCCAACACTGGGTCCT	AATCCGCCCAAAGAGGTACT	226 bp	90.0 %	0.992
Cr-XTH-2	ATAGTCATGGAAGCCGGTTG	GCTTGTGGGAGGAGACTCTG	208 bp	102.4 %	0.998
Cr-PAE	TGTTTAGCCGATGCTGGTTAC	AGCCTTACACGAAGCAGACAA	129 bp	107.8 %	0.997
Cr-RGP	CCAACGAAAATCATCCGAGT	GTTCTTGATGTGCTGCTCCA	237 bp	103.0 %	0.999
Cr-PX-1	TTGGCCTCGGAGTTAATGAC	GGATCCACGTTTGTCTCGTT	109 bp	102.3 %	1.000
Cr-PX-2	ACCCACCAATGTGACCATTT	CTTGGGAGAGGAGATGCAAG	141 bp	100.7 %	0.999
Cr-PX-3	CCACCCGTGACGTTATCTCT	TGGAAGGATGGCTTTGGTAG	121 bp	102.9 %	0.996
Cr-GH5	AATAACCACGGATGGTTGGA	GCTGATGCTGTGCTTGGTTA	137 bp	95.6 %	0.993
Cr-GT20	CACCGAAGCAGACCAATACAT	CCGAGATTATACCCGGAACAT	241 bp	103.7 %	0.995
Cg-ACTIN	CCCATACCAACCATCACTCC	TGAGGATATTCAGCCCCTTG	138 bp	105.1 %	0.996
Cg-SF2	ACATCGCTCTCTCAGGGAAA	CATCAGCATCACGAGCATCT	205 bp	108.9 %	0.988
Cg-XTH-1	AGATGATGTGTTGGGGCTTC	TGGTGAGCCATACACTCTGC	137 bp	95.0 %	0.987
Cg-XTH-2	TAAGGCCAGATACCCCACAG	AAACATGAGCCCAATTCCAC	133 bp	102.4 %	0.997

Supplementary Tables S2 and S3 are presented in a separate Excel file.

Supplementary Figure S1

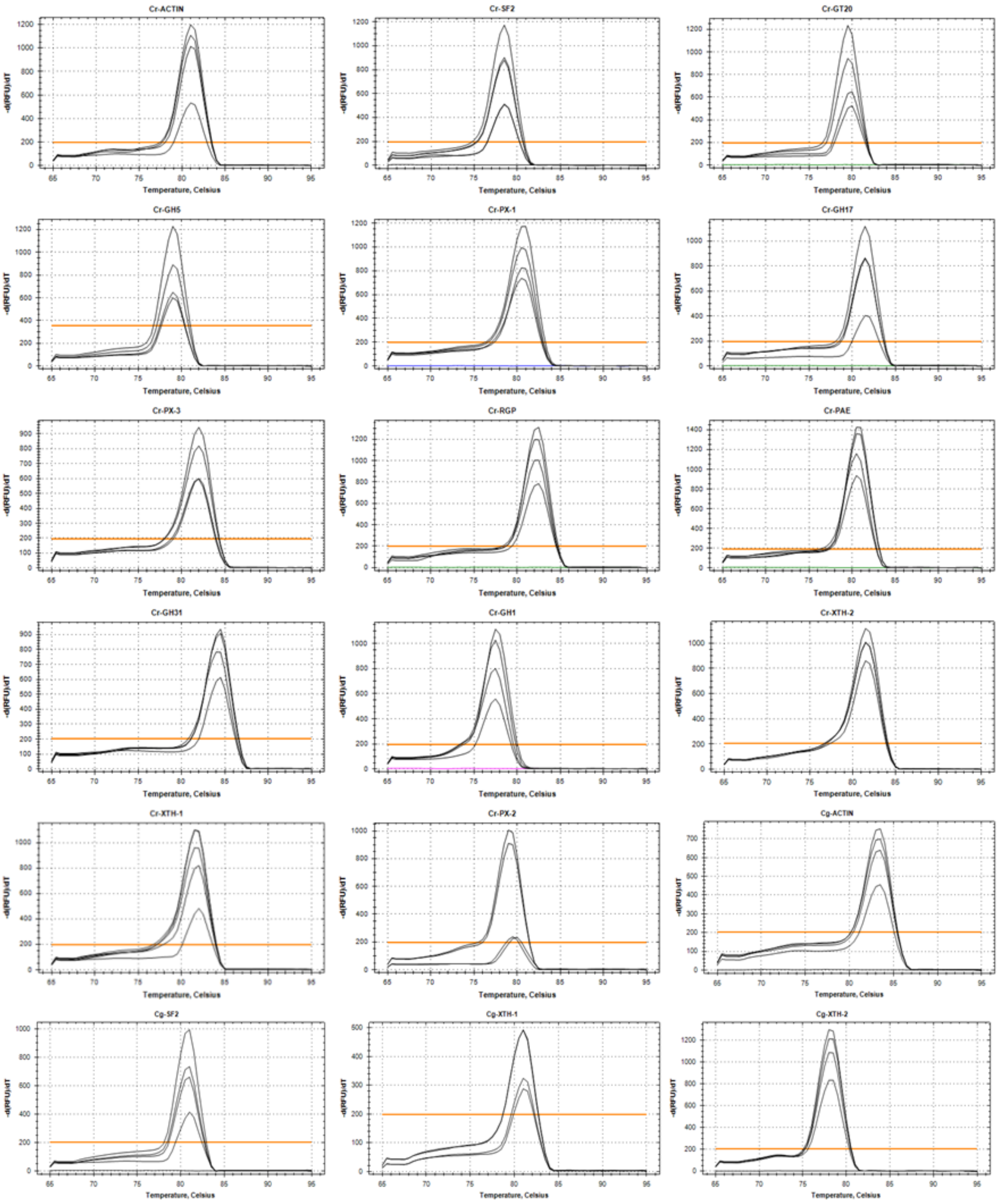

Suppl. Fig. S1. Melt peaks of qPCR amplicons. The negative derivative of the change in fluorescence intensity is plotted as a function of temperature.

Supplementary Figure S2

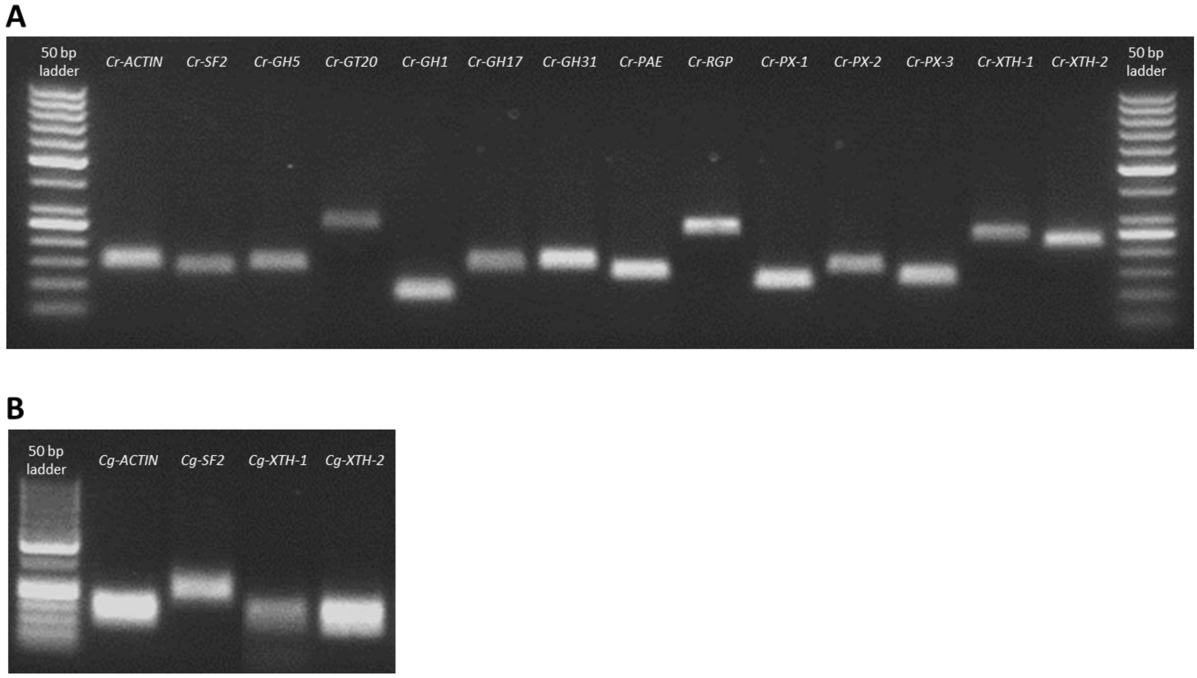

Suppl. Fig. S2. Size separation of (A) *C. reflexa* and (B) *C. gronovii* qPCR amplicons on 2 % agarose gels. GeneRuler 50 bp Ladder (Life Technologies) is used for size estimation.

Supplementary Figure S3

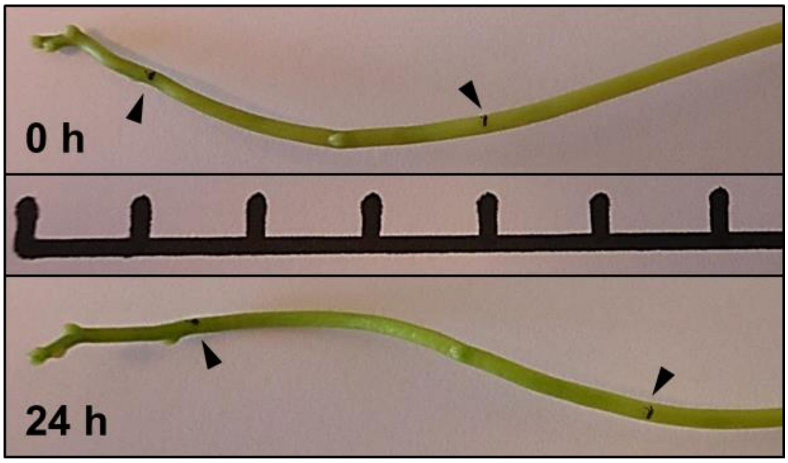

Suppl. Fig. S3. Stem elongation occurring in the region just below the apical shoot tip as indicated by increased distance between markings (arrowheads) after 24 hours. Distances between ruler marks are one cm.

Supplementary Figure S4

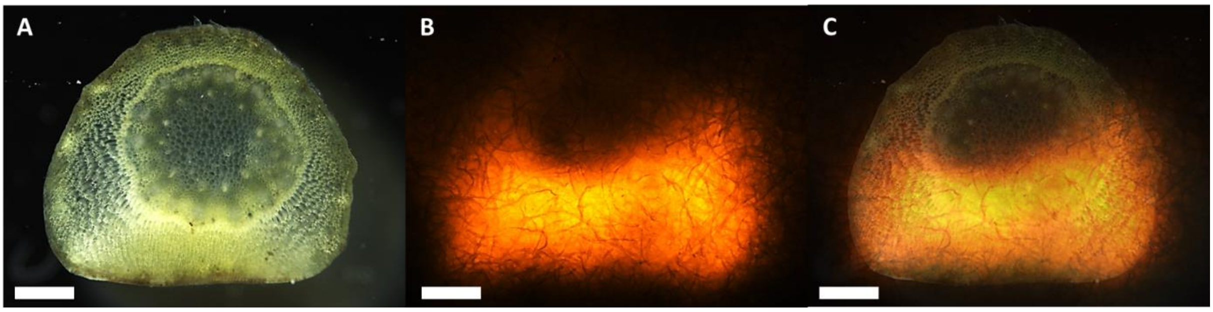

Suppl. Fig. S4. XET action in FR light-induced haustoria. (A) Cross-section of haustorium that was tissue printed on XET test paper three days after light treatment. (B) Fluorescence micrograph showing XET action in the printed tissue in A. (C) Merged picture of A-B. Scale bars are 500 µm. Note that the vein pattern in B and C comes from the fibres in the test paper.

Supplementary Figure S5

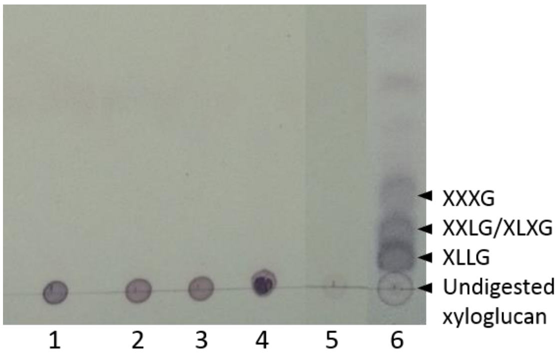

Suppl. Fig. S5. Hydrolytic activity of a haustorial enzyme extract from *C. reflexa* towards xyloglucan. Enzymes were extracted from *C. reflexa* haustoria by homogenizing plant material in extraction buffer (50 mM Na-acetate pH 5.5, 300 mM NaCl, 10 % glycerol, 10 % PVP40) and incubating on ice for two hours. Thin-layer chromatography of 2 µl sample-spots was executed on silica gel with n-butanol:acetic acid:water (3:1:1) as mobile phase. Carbohydrates on the chromatogram were detected by staining with the thymol-sulfuric acid reagent. Lane 1: tamarind xyloglucan only; Lanes 2, 3 and 4: incubation of tamarind xyloglucan with *C. reflexa* enzyme extract for zero, two and 24 hours at 25 °C, respectively; Lane 5: enzyme extract only; Lane 6: incubation of tamarind xyloglucan with a recombinant xyloglucanase (E-XEGP from Megazyme) at 25 °C. The four main xyloglucan oligosaccharide (XXXG, XXLG, XLXG and XLLG) hydrolysis products are marked with arrow tips.
